# Molecular Detection of Arboviruses in *Aedes* spp. Larvae During Entomo-Virological Surveillance in a Hospital Area of Brasília, Federal District, Brazil

**DOI:** 10.1590/0037-8682-0105-2026

**Published:** 2026-10-16

**Authors:** Renato Lemos Pereira, Kelliane Almeida de Medeiros, Marcelo Banho de Andrade Reis, Diane Sthefany Lima de Oliveira, Mércia Tayane Pereira dos Santos de Oliveira, Laysa Michelle Rodrigues Santos, Dalva Iara Ghilardi de Paula, Angela Rosa André, Marcelo Henrique Soller Ramada, Wanderson Kleber de Oliveira

**Affiliations:** 1 Hospital das Forças Armadas, Direção Técnica de Ensino e Pesquisa, Divisão de Pesquisa, Brasília, DF. Brasil.; 2 Universidade Católica de Brasília, Programa de Pós-Graduação em Ciências Genômicas e Biotecnologia, Águas Claras, DF, Brasil.; 3 Secretaria de Educação do Distrito Federal, Brasília, DF, Brasil.; 4 Universidade Católica de Brasília, Programa de Pós-Graduação em Gerontologia, Águas Claras, DF, Brasil.

**Keywords:** Ovitrap, RT-qPCR, One Health, Epidemiological monitoring, Entomo-virological surveillance

## Abstract

**Background:**

Arboviruses in hospital environments may represent a neglected risk of nosocomial infections. This study investigated arboviruses in *Aedes* spp. larvae collected from a hospital area in Brasília, Federal District, Brazil.

**Methods:**

Eighteen ovitraps were deployed for 26 weeks. Hatched *Aedes* spp. larvae were pooled weekly, and viral detection was performed by reverse transcription-quantitative PCR.

**Results:**

Dengue virus serotypes 1, 2, and 4, Zika virus, and Chikungunya virus were identified, with an overall positivity rate of 30.76%.

**Conclusion:**

The surveillance strategy effectively detected arboviral RNA in a hospital setting, suggesting local arboviral circulation associated with the transmission triad.

Arboviruses, including *Dengue*, *Zika*, and *Chikungunya*, represent a global public health threat, particularly in Brazil, where climate change has contributed to their emergence and changing distribution. Dengue alone causes approximately 390 million infections annually worldwide^1^. In Brazil’s Federal District, dengue is hyperendemic, with continuous circulation of all four serotypes. Vertical transmission in *Aedes* spp. larvae contributes to viral persistence and may facilitate outbreak prediction, while high mosquito infestation levels in healthcare facilities represent a neglected risk of nosocomial transmission^2^ ^,^ ^3^.

Current prevention strategies focus mainly on vector control, despite the limitations of entomological surveillance performed without virological monitoring^4^. Entomo-virological surveillance integrates vector and pathogen detection, enabling early detection of outbreaks and improved control strategies^4^ ^,^ ^5^.

Strategic surveillance points with intense human movement, including ports, airports, and sentinel sites, provide critical information for predicting outbreaks. Health facilities represent key “hotspots” where vectors, hosts, and viruses converge. However, nosocomial arbovirus transmission remains largely overlooked. Environmental surveillance under a One Health framework supports early detection of vector and pathogen spread, facilitating timely public health responses^5^ ^,^ ^6^.

Entomo-virological monitoring at neglected strategic locations, such as hospitals, offers actionable insights into transmission risk and supports targeted interventions. Ovitrap-based surveillance of *Aedes* spp., combined with reverse transcription-quantitative PCR (RT-qPCR), is a low-cost and effective approach to detect circulating arboviruses and strengthen health system preparedness.

Accordingly, the objective of this study was to implement entomo-virological monitoring using ovitraps and RT-qPCR to detect Dengue virus (serotypes 1-4), Zika virus, and Chikungunya virus in *Aedes* spp. larvae collected from a hospital area to predict and prevent arbovirus circulation.

The study area was located in Brazil’s capital, the Federal District (15º 48’ 04”S, 47º 56’ 06”W). The study site was a military area with controlled access containing residential complexes and two hospitals (the Armed Forces Hospital [AFH] and the Cardiology and Transplant Institute of the Federal District [CTIFD]). Rainfall in the region is distributed across a rainy season (Oct-Apr) and a dry season (May-Sept).

Human activity in the area is primarily associated with the hospitals. The AFH is a military hospital that provides medical-hospital care to Armed Forces beneficiaries and other affiliates, including civilian employees of the Ministry of Defense, embassies, and the Presidency of the Republic. The CTIFD is a private nonprofit referral center providing high-complexity cardiovascular care and transplants, serving patients from the Federal District and the North and Midwest regions.

Over 26 weeks, ovitraps were deployed at 18 locations around the hospitals and nearby residential areas, with spatial planning performed using QGIS and Google Earth (Figure 1) to ensure approximately 150-m spacing between traps, based on vector biology. Ovitraps consisted of 0.5-L black plastic containers with wooden paddles (3 × 12 cm) immersed in water and brewer’s yeast (0.04%). Paddles were labeled and installed with the rough side facing upward. Traps were inspected weekly and replaced every 7 days.

**FIGURE 1:**
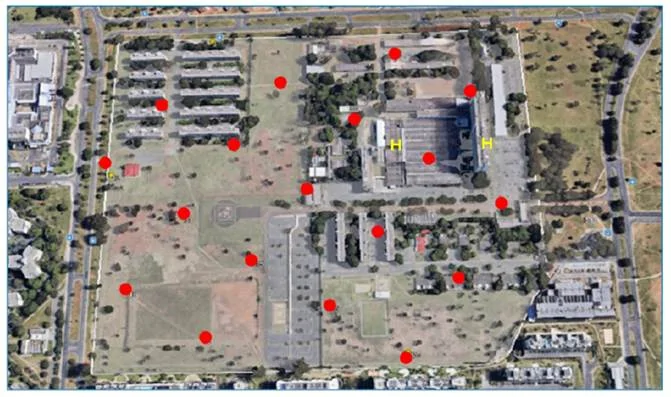
Map of the study area showing 18 ovitraps (red dots), two hospitals (H) and residential zones in an Administrative Region of Brasília, Federal District.

Weekly ovitrap paddles were placed in labeled bags and transported to the AFH Research Laboratory. Eggs were counted on the entire paddle surface using a stereomicroscope (20×) and a manual counter. The following entomological indices were calculated: Egg Density Index (EDI), Ovitrap Positivity Index (OPI), and Vector Density Index (VDI) (Supplementary Material Table 1). For hatching, paddles were submerged in water. Fourth-instar (L4) *Aedes* spp. larvae were grouped into pools of 10 in 1.5-mL microtubes, labeled by trap number and date, and stored at −80 °C until analysis.

For PCR analysis, the weekly samples from the 18 sites, each containing 10 larvae, were grouped into weekly pools. Larvae were homogenized in 140 µL of nuclease-free water with sodium azide (AVE buffer) in polypropylene microtubes. Viral RNA was extracted using the QIAamp Viral RNA Mini Kit (QIAGEN, USA) on a QIAcube Connect automated extractor, following the manufacturer's protocols. RT-qPCR was performed using an adapted IBMP Biomol ZDC Kit protocol for qualitative detection of ZIKV, DENV (serotypes 1-4), and CHIKV RNA in *Aedes* spp. larvae. The internal amplification control was omitted, as extraction and amplification procedures had been previously validated. Exogenous extraction and positive and negative controls were included to ensure assay performance. Negative results were interpreted cautiously owing to potential residual inhibition. Amplification was performed on a CFX96 Touch Real-Time PCR System (Bio-Rad, USA). Samples were considered positive when typical amplification curves were observed for ZIKV, CHIKV, or DENV targets with Ct values <36. A preliminary optimization phase was conducted using a convenience subset of larvae from weekly collections. Positive findings from this validation step were considered reliable and incorporated into the final analysis.

Statistical analysis was performed using GraphPad Prism 10.6.0 (GraphPad Software, Boston, MA, USA), applying simple linear regression (Ordinary Least Squares) and reporting slope, intercept, standard error (SE), 95% confidence intervals (CIs), R², Sy.x, and the overall F-test (DFn = 1, DFd = 24) to test whether the slope ≠ 0, adopting α = 0.05 (two-tailed). Model assumptions were assessed using residual inspection (random dispersion and homoscedasticity) and scatter plots to verify linearity.

This entomo-virological study involved only invertebrate species (*Aedes* sp.). Therefore, it was exempt from ethical approval for human research (Law 14.874/2024) and animal research (Law 11.794/2008) under Brazilian regulations.

According to INMET data for the study period (September 19, 2019, to March 26, 2020), the second half of 2019 was marked by the vigorous return of rainfall beginning in November, whereas the first half of 2020 exhibited a rainy-summer pattern concentrated between January and March^7^.

A total of 34,564 eggs were collected from the 18 traps during the 26 weeks. Weekly monitoring revealed a progressive increase in egg numbers over time, consistent with the onset of the rainy season in the region, peaking in week 14 (Epidemiological Week 52/2019; Figure 2A). Figure 2B shows the OPI increased consistently from week 10 (Epidemiological Week 48/19), with an upward trend of 2.07%/week (95% CI: 1.153-2.982; SE = 0.4432; intercept = 37.69%, 95% CI: 23.57-51.82; R² = 0.4755; Sy.x = 16.95; F(1,24) = 21.76; *p* < 0.0001; mean OPI: 65.6%; median = 75%; maximum = 88.9% in week 10). VDI and EDI values showed increasing oviposition intensity, peaking in week 14. VDI increased by 3.469 eggs/trap/week (95% CI: 1.770-5.167; SE = 0.8230; intercept = 27.02, 95% CI: 0.793-53.25; R² = 0.4253; Sy.x = 31.47; F(1,24) = 17.76; *p* = 0.0003; mean VDI = 73.9 eggs/trap; median = 79.7; maximum = 156.8). EDI increased by 3.326 eggs/positive trap/week (95% CI: 1.546-5.106; SE = 0.8624; intercept = 57.91, 95% CI: 30.42-85.40; R² = 0.3825; Sy.x = 32.98; F(1,24) = 14.87; *p* = 0.0008; mean EDI = 102.8 eggs/positive trap; median = 103.1; peak = 201.6 in week 14). Collectively, the three models indicate a concomitant increase in distribution (OPI) and intensity (VDI/EDI) of oviposition across the 18 traps.

**FIGURE 2:**
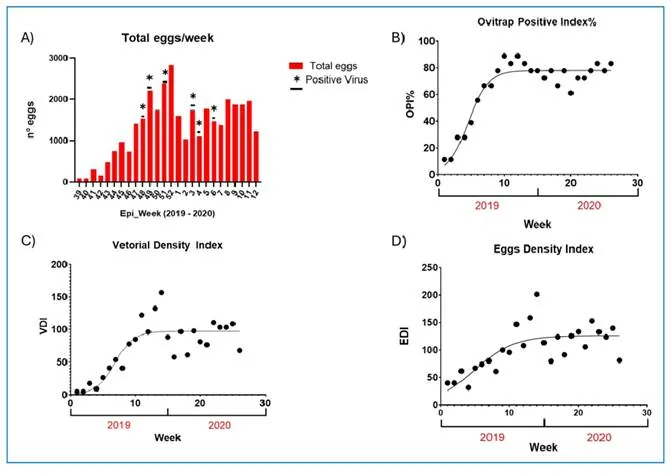
There is a consistent increase in oviposition intensity, demonstrated by the rising number of eggs per epidemiological week **(A)**; a consistent increase in mosquito dispersion, as shown by the rising Ovitrap Positivity Index (OPI) **(B)**; the increase in the Vector Density Index (VDI) **(C)**; and the increase in the Egg Density Index (EDI) **(D)**. In panel A (*), the detection of arboviruses in larvae hatched from collected eggs is highlighted beginning in epidemiological week 48, including the detection of *Zika virus*, *Chikungunya virus*, and *Dengue virus* serotypes 1, 2, and 4.

Figure 2A highlights the detection of DENV-2 (Epidemiological Week 48/19), DENV-1 (Week 51/19 and 06/20), DENV-4 (Week 51/19 and 06/20), ZIKV (Week 49/19 and 04/20), and CHIKV (Week 03/20) by RT-qPCR in larvae hatched from eggs collected using ovitraps. Table 1 presents the quantification cycles (Cq) for the target regions in positive samples. The positivity rate among the 26 pools analyzed was 30.76%.

**TABLE 1:** Positive results obtained by real-time RT-qPCR.

Virus	RT-qPCR		Epidemiology	
	Abs (260/280 nm)	Cq	Epidemiological Week	Positivity Index*
DENV 2	2.1	13	48/2019	3.84%
DENV 1	2.38	17	51/2019	7.69%
DENV 1	2.06	19	06/2020	
DENV 4	2.38	19	51/2019	7.69%
DENV 4	2.06	15	06/2020	
ZIKV	2.14	22	49/2019	7.69%
ZIKV	2.18	16	04/2020	
CHIKV	1.97	29	03/2020	3.84%

Positive results obtained by reverse transcription-quantitative PCR **(RT-qPCR)**, including quantification cycle (Cq) values and absorbance **(Abs)** measurements after sample extraction. Results are presented according to the epidemiological week of detection and the positivity index (number of positive pools/total number of pools × 100). *Total positivity index: 30.76%. **ZIKV:** Zika virus; **CHIKV:** Chikungunya virus; **DENV:** Dengue virus.

This study demonstrated the potential of entomo-virological surveillance to detect arbovirus circulation in a hospital setting. Using ovitrap-based monitoring and RT-qPCR analysis of hatched larvae, Zika, Chikungunya, and Dengue virus serotypes 1, 2, and 4 were identified during the pre-epidemic phase of the 2019-2020 seasonal cycle, highlighting the value of strategic hospital-based surveillance in areas with high human activity.

During the annual endemic cycle, healthcare facilities may concentrate the essential transmission triad-infected patients, susceptible individuals, and vectors^4^. This may make hospital environments high-risk potential convergence zones for nosocomial transmission and strategic sites for early detection of viral circulation^4^ ^,^ ^8^. Because of high levels of human movement and vector presence, hospitals may function as sentinel sites, similar to ports and airports. In this study, a high positivity rate and detection of multiple arboviruses in a single area reinforce their epidemiological relevance^4^. Population heterogeneity among patients served by the two hospitals may account for the high positivity rate and viral diversity observed. Integrating entomo-virological and genomic surveillance may improve near-real-time prediction and response^8^.

Another relevant factor is that the convergence of transmission determinants may represent a risk to both patients in the two hospitals (in the context of healthcare-associated infections) and for healthcare workers (through occupational exposure). The concept that health facilities should receive differentiated attention as surveillance areas is supported by various authors^4^ ^,^ ^5^ ^,^ ^8^.

A strategic advancement in arbovirus surveillance is the integration of entomological and virological monitoring. Unlike conventional systems based on vector indices or human case reports, entomo-virology (xenomonitoring) enables early detection of viral circulation directly in vectors, thereby transforming ovitraps into early warning tools^9^ ^-^ ^11^. A single field effort generates complementary entomological and virological data streams, providing information on vector density and pathogen circulation^12^. This integrated approach enabled the detection of five viruses during peak oviposition weeks (Figure 2A), with a positivity rate of 30.76%, reinforcing the association between vector activity and viral circulation.

In the present study, the DENV-4 serotype was detected in the analyzed samples, indicating environmental viral circulation in the investigated area. This finding is not consistent with official data from the Federal District. During 2019 and 2020, DENV-4 was not laboratory-confirmed in patient samples analyzed by the Central Public Health Laboratory of the Federal District (LACEN-DF). According to epidemiological reports, only DENV-1 and DENV-2 were identified during those two years. In total, 1,698 clinical samples were analyzed during this period (755 classified as DENV-1 and 943 as DENV-2), with no detections of DENV-3 or DENV-4. Thus, the DENV-4 detection in *Aedes* larvae suggests that environmental viral circulation may not have been captured by conventional clinical surveillance since 2016, reinforcing the potential of entomo-virological monitoring to identify serotypes circulating silently in the environment at an early stage^2^ ^,^ ^13^ ^,^ ^14^.

Another relevant finding was the detection of viral RNA in larvae, indicating vertical transmission among arthropod vectors^3^. Mosquito infection may have originated either from endemic circulation during egg collection or from previous transmission cycles owing to viral persistence in eggs. *Aedes* spp. eggs can withstand harsh environmental conditions and persist through interepidemic periods. Both mechanisms are plausible under the conditions of this study, as sampling occurred at the onset of the 2019-2020 epidemic cycle^6^.

From a One Health perspective, understanding vector-mediated viral persistence, particularly in eggs, is critical for early detection of transmission. This mechanism may allow arboviruses to sustain silent circulation during interepidemic periods. Asymptomatic humans and wild hosts may also contribute to viral maintenance, creating an enzootic and resilient nature that challenges control efforts. These dynamics underscore the need for integrated surveillance of vectors, human hosts, and animal reservoirs^3^ ^,^ ^6^ ^,^ ^8^.

However, structuring this type of surveillance requires laboratory infrastructure and specialized technical staff. Another challenge is the absence of diagnostic kits specifically developed for entomological use^15^. In this study, a commercial diagnostic kit designed for human blood samples was adapted, posing challenges related to Cq/Ct interpretation, sample quality, and assay sensitivity.

The entomo-virological surveillance strategy implemented in hospital areas, first applied in the Federal District, proved effective for detecting arboviral RNA indicative of local viral activity, including Dengue, Zika, and Chikungunya, supporting its value for surveillance and prevention of healthcare-associated infections. Combined ovitrap and RT-qPCR analyses provided molecular evidence suggestive of potential vertical transmission, highlighting the vector’s potential role in maintaining viral circulation during interepidemic cycles. The detection of multiple viruses, including Dengue virus serotype 4, which had not been detected by conventional clinical surveillance in the Federal District since 2016, further suggests that hospital environments may represent settings of heightened transmission concern owing to convergence of the transmission triad.

## Data Availability

Research data available in the body of the article.
