# Supplementary material for: Molecular Detection of Arboviruses in Aedes spp. Larvae During Entomo-Virological Surveillance in a Hospital Area of Brasília, Federal District, Brazil
Source: Rev Soc Bras Med Trop. 2026 Oct 16;59:e0105-2026. doi: 10.1590/0037-8682-0105-2026 (PMC13674194; doi:10.1590/0037-8682-0105-2026)
Supplement: Supplementary file 1 [file 1678-9849-rsbmt-59-e0105-2026-md1.pdf]

Supplementary Material Table 1

Week	Eggs		Entomological index		
	Positive Traps (n)	Total Eggs (n)	EDI (n)	IPO (%)	VDI (n)
1	2	80	40.0	11.1	4.4
2	2	80	40.0	11.1	4.4
3	5	310	62.0	27.8	17.2
4	5	158	31.6	27.8	8.8
5	7	471	67.3	38.9	26.2
6	10	740	74.0	55.6	41.1
7	12	965	80.4	66.7	53.6
8	12	735	61.3	66.7	40.8
9	14	1403	100.2	77.8	77.9
10	16	1532	95.8	88.9	85.1
11	15	2202	146.8	83.3	122.3
12	16	1732	108.3	88.9	96.2
13	15	2383	158.9	83.3	132.4
14	14	2822	201.6	77.8	156.8
15	14	1591	113.6	77.8	88.4
16	13	1036	79.7	72.2	57.6
17	14	1738	124.1	77.8	96.6
18	12	1097	91.4	66.7	60.9
19	14	1761	125.8	77.8	97.8
20	11	1466	133.3	61.1	81.4
21	13	1378	106.0	72.2	76.6
22	13	1990	153.1	72.2	110.6
23	14	1860	132.9	77.8	103.3
24	15	1860	124.0	83.3	103.3
25	14	1956	139.7	77.8	108.7
26	15	1218	81.2	83.3	67.7

Weekly quantification of eggs collected from ovitraps. After recording the positive traps and counting the eggs. the following entomological indices were calculated: I – Egg Density Index (EDI) *(Total number of eggs / Number of positive paddles)*: used for the early detection of new infestations; II – Ovitrap Positivity Index (OPI) *(Total number of positive ovitraps / Total number of examined ovitraps × 100)*: used to determine the spatial distribution of the vector in the evaluated area; and III – Vector Density Index (VDI) *(Total number of eggs found / Number of examined paddles)*: used to evaluate the abundance of vectors engaged in reproductive activity.
